# *NtCIPK9*: A Calcineurin B-Like Protein-Interacting Protein Kinase From the Halophyte *Nitraria tangutorum*, Enhances Arabidopsis Salt Tolerance

**DOI:** 10.3389/fpls.2020.01112

**Published:** 2020-08-21

**Authors:** Lu Lu, Xinying Chen, Liming Zhu, Mengjuan Li, Jingbo Zhang, Xiuyan Yang, Pengkai Wang, Ye Lu, Tielong Cheng, Jisen Shi, Yin Yi, Jinhui Chen

**Affiliations:** ^1^Key Laboratory of Forestry Genetics & Biotechnology of Ministry of Education, Co-Innovation Center for Sustainable Forestry in Southern China, Nanjing Forestry University, Nanjing, China; ^2^Experimental Center of Desert Forestry, Chinese Academy of Forestry, Dengkou, China; ^3^Research Center of Saline and Alkali Land of National Forestry and Grassland Administration, China Academy of Forestry, Beijing, China; ^4^State Forestry Administration Key Laboratory of Biodiversity Conservation in Karst Mountainous Areas of Southwestern China, Guizhou Normal University, Guiyang, China; ^5^Guizhou Provincial Key Laboratory of Plant Physiology and Developmental Regulation, Guizhou Normal University, Guiyang, China

**Keywords:** halophyte, *Nitraria tangutorum*, *CIPK9*, ion homeostasis, salt tolerance

## Abstract

Calcineurin B-like protein-interacting protein kinases (*CIPKs*) play essential roles in plant abiotic stress response. In order to better understand salt tolerance, we cloned and analyzed the *NtCIPK9* gene from the halophyte *Nitraria tangutorum*. Phylogenetic analysis shows that *NtCIPK9* belongs to a sister clade with the *Arabidopsis AtCIPK9* gene and is thought to localize to the plasma membrane. *NtCIPK9* shows the highest expression level in the *Nitraria tangutorum* root under normal growth conditions, whereas after NaCl treatment, the highest expression was found in the blade. *NtCIPK9*-overexpressing Arabidopsis plants have a higher seed germination rate, longer root length, and displayed higher salt tolerance than wild type seedlings under salt stress conditions. Furthermore, *NtCIPK9* overexpression might enhance the expression of genes related to K^+^ transportation after NaCl treatment. Thus, we conclude that *NtCIPK9* increases transgenic plant salt tolerance and reduces damage associated with salt stress by promoting the expression of genes controlling ion homeostasis. Our results suggest that *NtCIPK9* could serve as an ideal candidate gene to genetically engineer salt-tolerant plants.

## Introduction

Soil salinity gradually accumulates along with global environmental degradation and limits the quality and productivity of most important agricultural crops and trees worldwide (Mahajan and Tuteja, 2005; Zhou et al., 2014; Guo et al., 2018). Currently, soil salinization is estimated to result in one-third of the world’s limited arable land loss, and will increase to 50% by the middle of 21st century (Zhu et al., 1998; Wang et al., 2003). Thus, improving the adaptability of plants to salinized soil is crucial not only for plant survival, but also for effective soil utilization. Genetic engineering to improve the salt tolerance of crops has been actively investigated by plant scientists worldwide (Wang et al., 2003; Hu et al., 2005; Ashraf and Akram, 2009).

*Calcineurin B-like proteins* (*CBLs*) and their targets *CBL-interacting protein kinases* (*CIPKs*) are involved in the Ca^2+^ signal pathway that functions during stress response (Kudla et al., 1999; Shi et al., 1999; Kim et al., 2000); they play important roles in maintaining cytoplasm ion homeostasis and improving salt tolerance. *CIPKs* contain a typical protein kinase domain with a putative activation loop and a unique C-terminal regulatory region with a conserved NAF/FISL motif, both of which are necessary and sufficient for the function of these genes (Shi et al., 1999; Albrecht et al., 2001; Guo et al., 2001). *CIPKs* have been identified in many plant species (Albrecht et al., 2001; Kolukisaoglu et al., 2004; Lyzenga et al., 2013; Zhang et al., 2014), and remarkable progress has been made in exploring the functions of *CIPKs* (Zhu et al., 1998; Halfter et al., 2000; Shi et al., 2000; Qiu et al., 2002; Cheong et al., 2003; Chinnusamy et al., 2004; Sánchez-Barrena et al., 2005; Luan, 2009). In Arabidopsis, *CIPKs* affect cellular ion homeostasis under saline conditions by regulating ion transporters, such as *HKT1* (*HIGH-AFFINITY K^+^ TRANSPORTER1*). *HKT1* improves salinity tolerance by removing Na^+^ from the transpiration stream and promoting the absorption of K^+^ in *Arabidopsis* (Kato et al., 2001; Cheong et al., 2003; Li et al., 2006; Xu et al., 2006; Grefen and Blatt, 2012). In addition, *CIPKs* also regulate the expression of stress-responsive genes mediated by the abscisic acid pathway during seed germination and at the seedling stage (Kim et al., 2003; Pandey et al., 2008a; Piao et al., 2010). *CIPK* function is highly conserved across plant families, shown by studies in apple and tomato (Hu et al., 2016), *Cicer arietinum* (Tripathi et al., 2009), *Hordeum brevisubulatum* (Li et al., 2012), and *Nitraria tangutorum* (Himabindu et al., 2016). However, most studies have been performed in salinity sensitive plants (glycophytes), such as Arabidopsis, rice, and maize (Kolukisaoglu et al., 2004; Zhao et al., 2009; Rigó et al., 2016), limiting our understanding of how plants may adapt to saline environments. Therefore, studying how halophyte *CIPKs* homologs function might provide crucial perspective in addressing this question, as these genes could be functionally more efficient than their glycophyte counterparts (Himabindu et al., 2016).

*Nitraria tangutorum* Bobr. (a halophyte), is a shrub that belongs to the family Nitrariaceae Nitraria in Sapindales (Zhao et al., 2002; Chase and Reveal, 2009; Group, 2016; Lu et al., 2018), and is widely distributed in China’s northwestern region (Wang et al., 2014). This typical plant has a strong adaptability to high salinity, arid or semiarid environments. It can efficiently alleviate the degree of soil salinity–alkalinity, which could improve the utilization of saline areas and prevent soil desertification (Zhao et al., 2002; Yang et al., 2010a; Jie et al., 2011). Due to its ecological effect, studies on *Nitraria tangutorum* have mainly focused on the physiological and biochemical aspects of its adaptive mechanisms to abiotic stresses (Yang et al., 2010a; Yang et al., 2010b; Yang et al., 2012; Yang et al., 2013). Although *NtCIPK2* and *NtP5CS* from *Nitraria tangutorum* have been cloned and analyzed to a certain extent (Zheng L. et al., 2014; Zheng L. L. et al., 2014), there is a current lack of knowledge on how *Nitraria tangutorum* responds molecularly to salt stress. In order to reveal the functional genes supporting *Nitraria tangutorum* to deal with high salinity and promote the application of these functional genes from halophyte to glycophyte, in our study, we used rapid amplification of cDNA end (RACE) cloning to identify a novel *Nitraria tangutorum CIPK* gene, which shows significant homology to *Arabidopsis CIPK9*. Therefore, we named it *NtCIPK9* (*Nitraria tangutorum CIPK9*). Quantitative PCR analysis showed that *NtCIPK9* positively responds to 500 mM NaCl treatment in both the root and leaf of *Nitraria tangutorum*. We overexpressed *NtCIPK9* in Arabidopsis and compared the different abilities of salt resistance between transgenic plants and wild type plants. *NtCIPK9* overexpressing-plants displayed a higher germination efficiency, longer root length, more leaves, and a lower death rate than the wild type under salt stresses. The high K^+^ content and *AtHKT1* expression level in transgenic seedlings suggest that *NtCIPK9* enhanced salt tolerance by regulating expression of genes controlling ion homeostasis.

## Materials and Methods

### Plant Materials and Treatments

#### Nitraria tangutorum

*Nitraria tangutorum* seeds, provided by the Experimental Center for Desert Forestry of the Chinese Academy of forestry, were kept in sand with around 7% water at 4℃ for four weeks. Seeds were germinated in pots containing a mixture of soil and sand (1:1 ratio) at 26°C to 28°C and 16-h light/8-h dark cycle condition. The humidity of the chamber for plant growth was 55% to 60%. 6-month-old *Nitraria tangutorum* plants were irrigated with 500 mM NaCl and harvested at 0, 1, and 2 h after treatment for RNA extraction.

#### Arabidopsis thaliana

The *Arabidopsis thaliana* Columbia ecotype was used for *NtCIPK9* gene transformation through the floral dip method (Clough and Bent, 1998). Positive transgenic plants were selected using 50 mg/L Kanamycin and confirmed by PCR. Wild type and transgenic plants were grown under identical growth conditions in parallel. Seeds were harvested at the same time for phenotypical analysis. Arabidopsis seeds were sown on ½MS with 0, 100, and 150 mM NaCl for germination rate analysis, phenotypical observation, and ion content measurements. Three biological replicates were used for germination rate analysis. Each biological replicate included three technical replicates. At least 200 seeds per line have been used. WT and transgenic plants were sown on the same plates. 10-day-old seedlings were transferred to ½MS with 0, 100, and 150 NaCl for another 10 days to analyze survival rate of transgenic plants, three biological replicates for each experiment. Seedlings from 0 and 100 mM NaCl treatment were frozen in liquid nitrogen and stored at −80°C for qPCR experiments. *NtCIPK9* transgenic T2 heterozygous seeds have been sown in the pots. After four weeks, seedlings in pots were watered by 200 mM NaCl for 4 days. Three biological replicates and four experimental repeats have been conducted.

### Gene Cloning

Total RNA was extracted from *Nitraria tangutorum* leaves using a Total RNA Purification Kit (NORGEN, Thorold, ON, Canada), followed by removal of genomic DNA contamination using DNaseI (TaKaRa, Japan). Total RNA concentration and integrity were quantified by ultraviolet spectrophotometry and electrophoresis, respectively. First-strand cDNA was synthesized using reverse transcriptase (Invitrogen, Carlsbad, USA). Degenerate primers to amplify the *CIPK* fragment were designed based on the homeodomain of *CIPK* from poplar (Chen et al., 2011) and are listed in **Supplementary Table 1**. The full length sequence was obtained by 5’ and 3’-RACE (rapid amplification of cDNA ends) according to the SMARTer™ RACE cDNA Amplification Kit User manual (BD Bioscience Clontech, USA). Primers for RACE are listed in **Supplementary Table 2**. The amplified PCR product was purified and cloned into pMD19-T (TaKaRa, Japan) and sequenced (GenScript, Nanjing, China). After assembly, the complete *NtCIPK9* sequence was amplified from cDNA using the primers mentioned in **Supplementary Table 3**. To confirm whether the *NtCIPK9* genomic region also contains introns, we amplified *NtCIPK9* from *Nitraria tangutorum* genomic DNA using the same primers (**Supplementary Table 3**).

### Bioinformatics Analysis

The *NtCIPK9* homolog was identified by using NCBI blastp. Multisequence alignment was performed using DNAMAN 6.0 software (Lynnon Biosoft, Quebec, Canada). Conserved domains of *NtCIPK9* were predicted using InterProScan online software (http://www.ebi.ac.uk/InterProScan/). Phylogenetic trees were constructed with amino acid sequences of *NtCIPK9* and 26 Arabidopsis *CIPK* proteins using the Neighbor-joining method with 1,000 bootstrap replications and the Jones-Taylor-Thornton model in Mega 6 software. Sequence accession numbers are listed in **Supplementary Table 4**. Hydrophobic analysis and transmembrane domain prediction of the *NtCIPK9* protein were performed using ProtScale (http://ca.expasy.org/tools/protscale.html) and the TMHMM server (http://www.cbs.dtu.dk/services/TMHMM/).

56 protein-coding genes from 46 chloroplast genomes (**Supplementary Table 5**) were selected for phylogenetic analysis of *Nitraria tangutorum*, *Vitis vinifera* as outgroup. Sequences alignment was performed using ClustalW. Each orthologous gene was trimmed with trimAl version 1.2 (Capella-Gutiérrez et al., 2009). The trimmed alignments were concatenated using SequenceMatriX version 1.7.8 (Vaidya et al., 2011). A nucleotide matrix of 44780 sites was then constituted for Maximum parsimony analysis by PAUP 4.0 (Swofford, 2002).

### Subcellular Localization Assay

The full-length coding region of *NtCIPK9* was cloned into vector *pJIT166:GFP* for subcellular localization analysis. The recombinant plasmids were bombarded into onion epidermal cells according to a previously described method (Yokoi et al., 2002) and followed by fluorescence detection using a ZEISS X-cite 120Q fluorescence microscope (ZEISS, Germany). Three technical replicates have been performed. The primers for construction of GFP tagged *NtCIPK9* are listed in **Supplementary Table 6**.

### Quantitative Real-Time PCR Analysis

Total RNA isolation and reverse transcription were performed as mentioned above. Quantitative real-time PCR was performed using a SYBR-Green PCR Mastermix on a LightCycler^®^480 real-time PCR detection system (Roche, Basel, Switzerland) according to the manufacturer’s instruction. Expression levels of target genes were normalized using the housekeeping gene actin in *Nitraria tangutorum* (Wang et al., 2012) and ubiquitin10 (UBQ10) in *Arabidopsis* (Geldner et al., 2009). Three technical replicates for three independent transgenic lines were carried out for real-time PCR. Sequence-specific primers were designed using Primer 3.0 and Oligo 7 and are listed in **Supplementary Table 7**.

### Cation Content Measurements

Twenty-day-old seedlings grown on ½MS with 0, 100, and l50 mM NaCl were collected, respectively, and washed three times with ddH_2_O, then dried at 80°C for 3 day. Harvested samples were digested with the HNO_3_-HClO_4_ method (Zhao et al., 1994). After acid digestion, samples were diluted to a total volume of 50 mL with ddH_2_O and kept in new tubes before analysis using flame atomic absorption spectrophotometry (FAAS) (Karpiuk et al., 2016). Three biological replicates were performed for each ion content test experiment. Three technical replicates were repeated for each biological replicate.

## Results

### Conserved Domain of NtCIPK9

**Figure 1:**
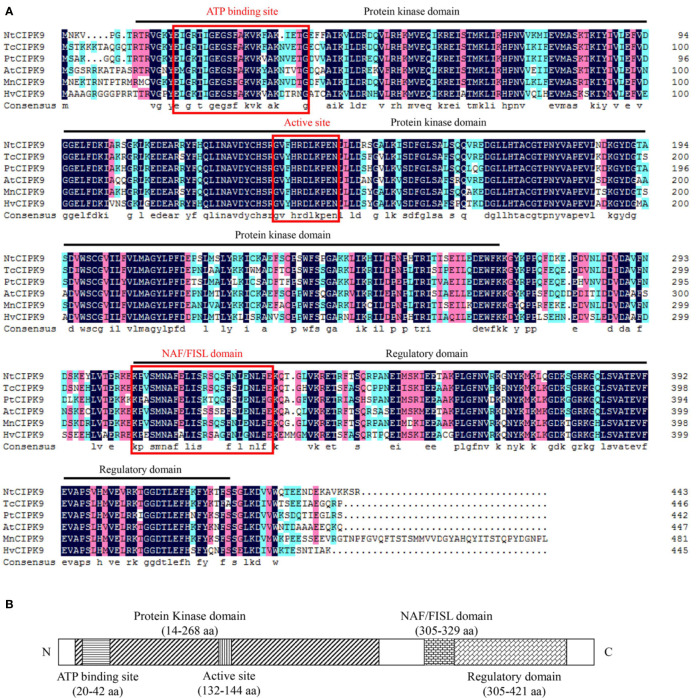
Multiple alignment and domain prediction of NtCIPK9. **(A)** Amino acid sequence alignment of NtCIPK9 with homologs from *Theobroma cacao* (EOX95608.1), *Populus trichocarpa* (XP_024450759.1), *Arabidopsis thaliana* (NP_171622.1), *Morus notabilis* (EXB83832.1), *Oryza sativa* (XP_015630713.1), *Hordeum vulgare* (AEZ51503.1), and *Zea mays* (PWZ05904.1). Dark blue shading indicates identical residues, and pink shading indicates similar residues. Dark lines demarcate the N-terminal kinase and C-terminal regulatory domains, while their active sites and NAF/FISH domain are marked with a red lines, the sequence identities between NtCIPK9 and others were shown at the end of the Multiple Alignment. **(B)** Schematic diagram of the domains of the NtCIPK9 protein. The amino acid position of the NtCIPK9 domain borders was predicted by InterProScan online software.

To start, we analyzed the basic properties of *NtCIPK9* sequence. Full length *NtCIPK9* is 1735 bp with a predicted open reading frame of 1332 bp nucleotides, a 5′UTR of 239 bp and a 3′UTR of 164 bp in length, encoding 443 amino acids with an estimated molecular weight 50.52 kDa. The acquired coding sequence shares high similarity with *CIPKs* from different plant species. The deduced NtCIPK9 protein sequence showed 82.18% identity with *Theobroma cacao* CIPK9 (TcCIPK9), 80% identity with *Populus trichocarpa* CIPK9 (PtCIPK9) and 77.78% identity with *Arabidopsis thaliana* CIPK9 (AtCIPK9) (**Figure 1A**). Consistent with other CIPKs, NtCIPK9 possesses an N-terminal SNF-1–related serine/threonine protein kinase domain (14–268 aa) and a C-terminal regulatory domain (305–421 aa) with a *CBL*-interacting NAF/FISL module (**Figure 1B**). Thus, this gene was designated as *NtCIPK9*, a novel member of the plant *CIPK* gene family.

**Figure 2:**
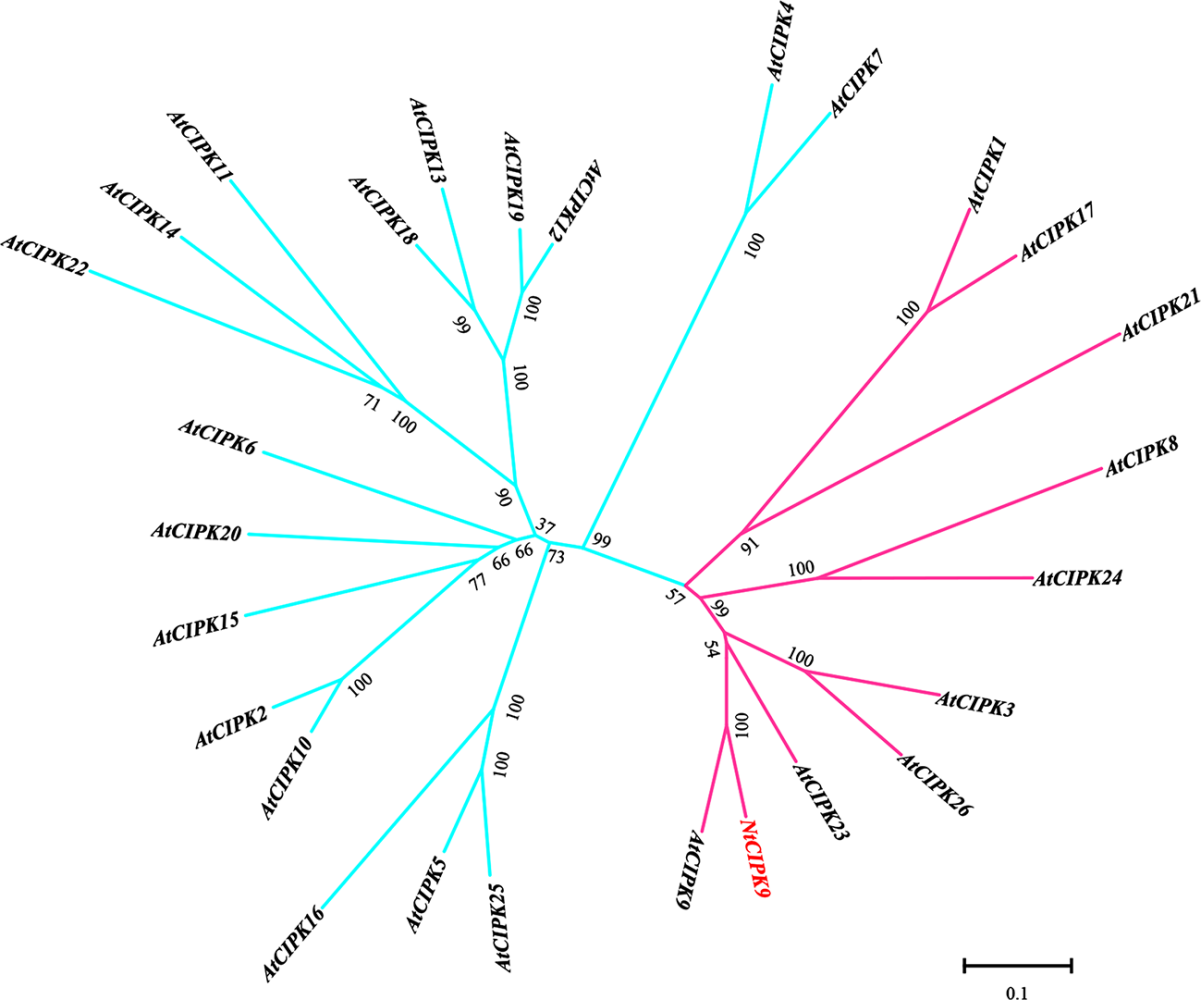
Phylogenetic analysis of NtCIPK9 with Arabidopsis CIPKs. The pink branch represents the subgroup of *CIPKs* with introns. The blue branch represents the clusters without intron.

Phylogenetic comparison of NtCIPK9 with the Arabidopsis CIPK family clustered NtCIPK9 as a sister branch of AtCIPK9 to the intron-rich subgroup (Yu et al., 2007) (**Figure 2**). To analyze whether the *NtCIPK9* gene contains introns, we amplified the genomic *NtCIPK9* sequence from genomic DNA. The result show genomic *NtCIPK9* harbors introns by DNA electrophoresis and sequencing (**Supplementary Figure 1A**), which is consistent with the results of our phylogenetic analysis. Besides, evolutionary study showed the *Nitraria tangutorum* was claded with *Sapindus mukorossi*, *Azadirachta indica*, *Zanthoxylum piperitum*, and *Citrus sinensis* in Sapindales (**Supplementary Figure 2**). *Citrus sinensis* is one of most important commercial fruit crops (Bausher et al., 2006).

### Subcellular Location of NtCIPK9

**Figure 3:**
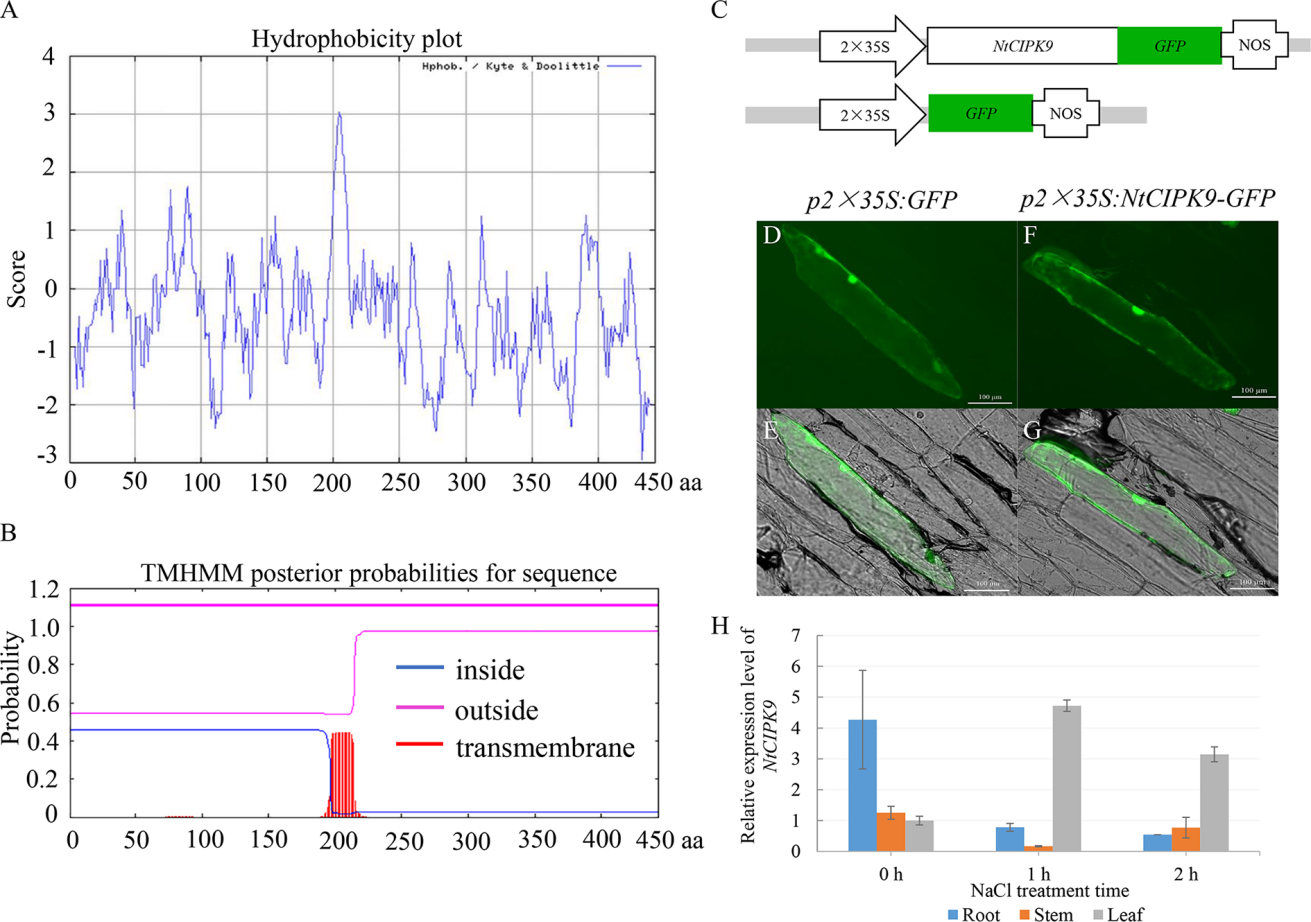
Structure analysis and subcellular localization of NtCIPK9. **(A)** Hydrophobicity plot of NtCIPK9. **(B)** The predicted transmembrane helix domain of NtCIPK9. **(C)** Schematic of the vectors used for analysis of NtCIPK9 subcellular localization. **(D–G)** NtCIPK9 subcellular localization. p35S:GFP serves as the control. **(H)** Relative expression level of *NtCIPK9* in different tissues of *Nitraria tangutorum*. Data represent means ± SD from three biological replicates.

A hydrophobicity blot indicated that the most hydrophobic segment of NtCIPK9 was located between amino acid residues 196 to 211 (**Figure 3A**), corresponding to the transmembrane domain predicted by the TMHMM Server 2.0 (**Figure 3B**). To further confirm the subcellular localization of NtCIPK9 in plant cells, a *35S:NtCIPK9*-*GFP* translational fusion was constructed with GFP tagged to the C-terminus of NtCIPK9 and *35S:GFP* was used as control (**Figure 3C**). The two vectors were bombarded into onion epidermal cells and transient expression of *NtCIPK9*-*GFP* was detected by epi-fluorescence. *35S:GFP* fluorescence was detected in the membrane and cytoplasm (**Figures 3D, E**), similar to the localization of NtCIPK9-GFP (**Figures 3F, G**). The hydrophobicity and subcellular location analysis suggest that NtCIPK9 might be one of membrane-bound proteins.

### *NtCIPK9* Responds to Salt Treatment in *Nitraria tangutorum*

To assess the expression of *NtCIPK9* in *Nitraria tangutorum* under salt stress conditions, we isolated total mRNA from different tissues (including root, stem, and leaf) after 2 h 500 mM NaCl treatment. qPCR results revealed that *NtCIPK9* showed relatively higher expression levels in the root than in the leaf and stem before salt treatment (**Figure 3H**). However, *NtCIPK9* transcription was upregulated in leaves after a 500 mM NaCl treatment (**Figure 3H**). Besides, the expression patterns in whole plants also showed the positive response of *NtCIPK9* to salt stress (**Supplementary Figure 1B** and **Supplementary Figure 3A**).

### Ectopic Expression of *NtCIPK9* in Arabidopsis Promotes Seed Germination Under Salt Stress

**Figure 4:**
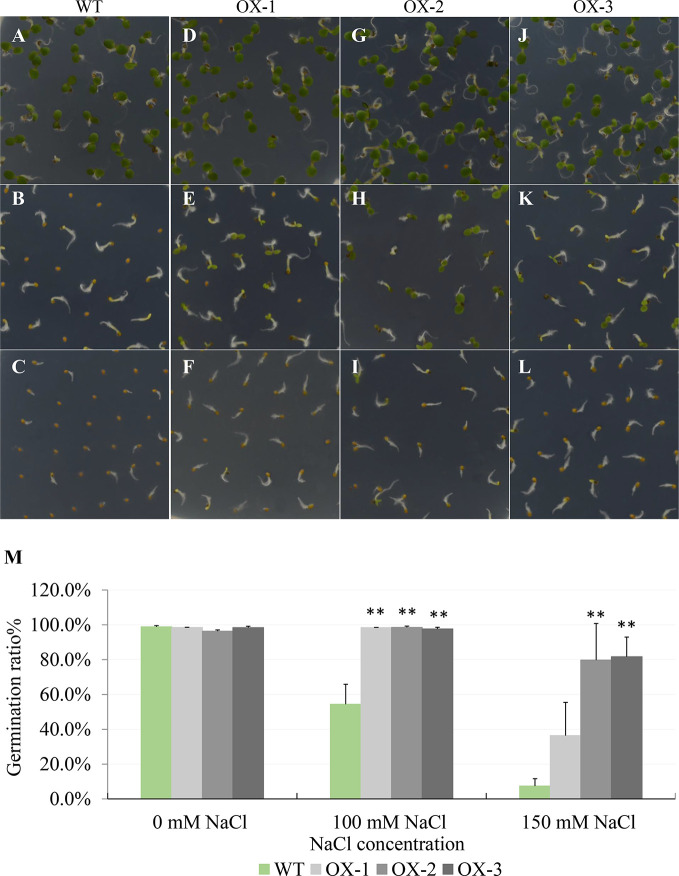
*NtCIPK9* promotes seed germination rate of Arabidopsis. **(A–C)** Wild type seeds germinated on ½ MS medium with 0, 100, and 150 mM NaCl respectively after 4 days. **(D–L)** Three independent transgenic lines of *35S:NtCIPK9*, depicted as OX-1, OX-2, and OX-3, germinated on different salt media as indicated. **(M)** Graphic of germination rate. Six technical replicates for each line were used in three biological replicates. **P < 0.01, ANOVA test was used for statistical analysis.

To further investigate how *NtCIPK9* affects salt tolerance, we overexpressed (*35S:NtCIPK9*) it in Arabidopsis. Seeds from three individual homozygous lines and WT were sown on ½ MS-agar plates to test their germination rate. On ½ MS without added NaCl, wild type and transgenic seeds showed no difference; yet on ½ MS with 100 and 150 mM added NaCl, 98.18% and 65.91% of *35S:NtCIPK9* seeds germinated, respectively, while only 54.39% and 7.42% of WT seeds germinated under the same conditions (**Figures 4A–L**). Therefore, we conclude that *NtCIPK9* significantly promotes seed germination under salt stress conditions (**Figure 4M**).

### Ectopic Expressing *NtCIPK9* Enhances Salt Tolerance in *Arabidopsis*

**Figure 5:**
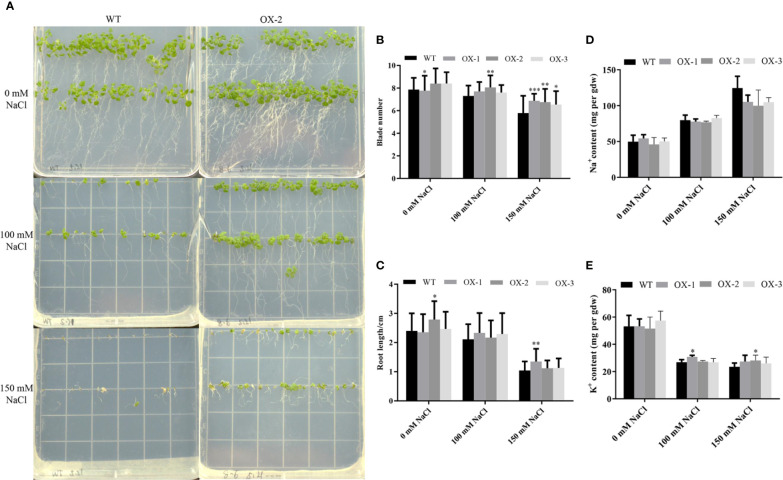
*NtCIPK9* transgenic plants show more vigorous growth than WT. **(A)** Transgenic plants grew faster 20 days post germination under different NaCl treatment conditions. **(B)** Quantification of plant leaf number. **(C)** Plant primary root length. At least 90 plants were analyzed for each line. **(D)** Na^+^ content of whole seedlings treated with 100 mM NaCl. **(E)** K^+^ content of whole seedlings treated with 100 mM NaCl. Three biological replicates and three technical replicates were performed for each ion content test experiment. ***P < 0.001; **P < 0.01; *P < 0.05. ANOVA test was performed for statistical analysis. Data represent means ± SD from three technical replicates.

**Figure 6:**
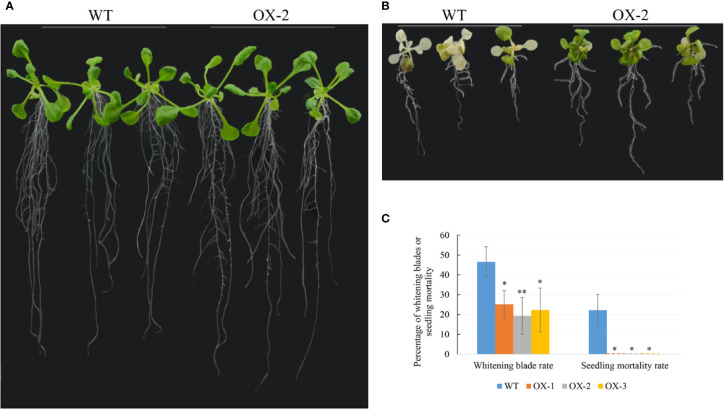
*35S:NtCIPK9* transgenic plants show a lower mortality rate than WT. **(A)** WT and transgenic plants grown on media without added NaCl for 20 days. **(B)** The phenotypes of WT and transgenic plants grown on media supplemented with 150 mM NaCl for 10 days. **(C)** The percentage of chlorotic leaves and mortality of plants. 18 plants were used for statistics by ANOVA, three biological replicates for each experiment. ANOVA test was used for statistical analysis. **P < 0.01; *P < 0.05.

**Figure 7:**
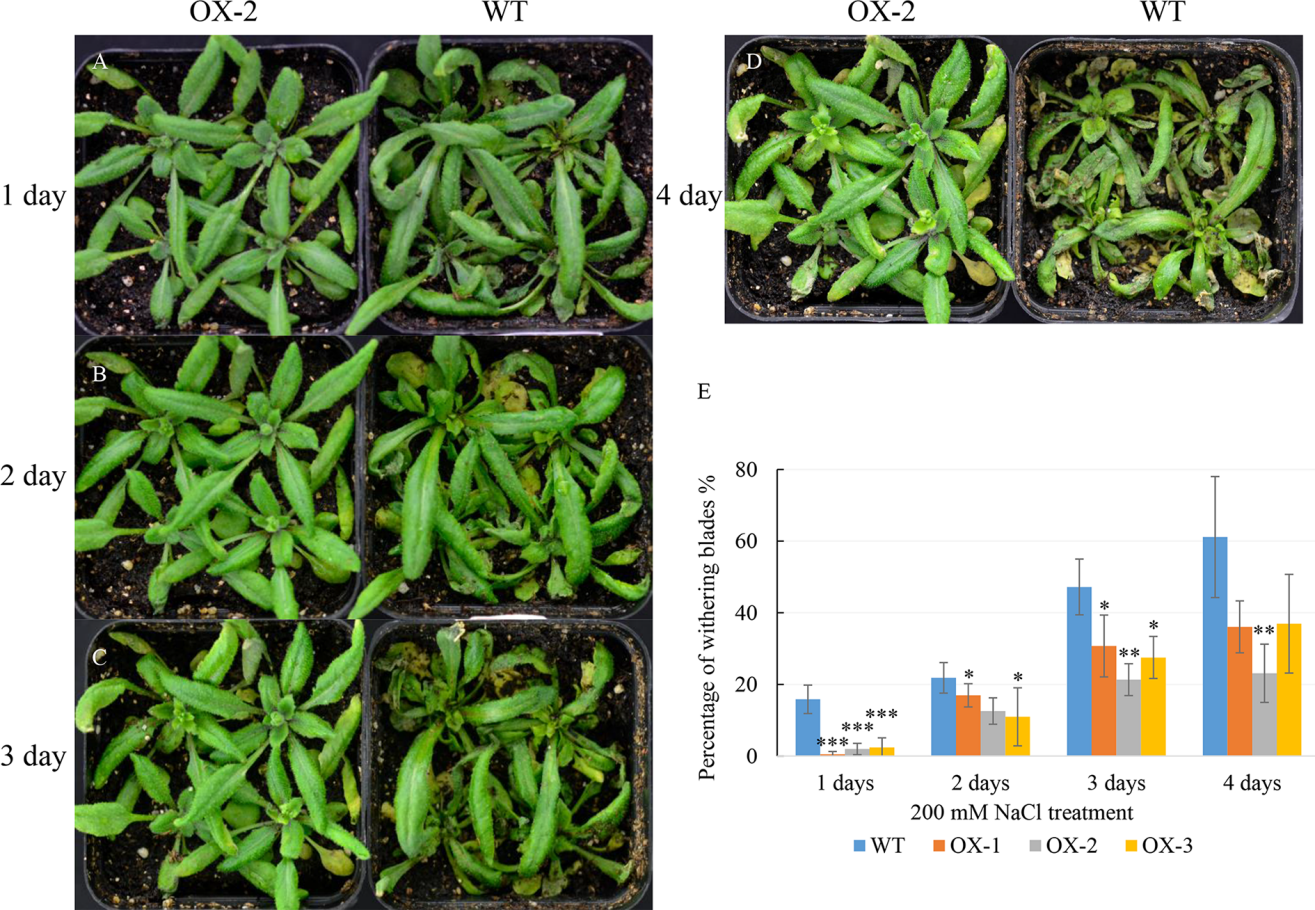
Improved salinity tolerance in heterozygous plants with *NtCIPK9* overexpression. **(A**–**D)** 200 mM NaCl treated 4-week-old T2 transgenic plants and WT in pots for four days respectively. **(E)** Percentage of withering leaves during salt treatment in pots. ANOVA test was used for statistical analysis. ***P < 0.001; **P < 0.01; *P < 0.05. Four experimental repeats have been used in three independent replicates.

**Figure 8:**
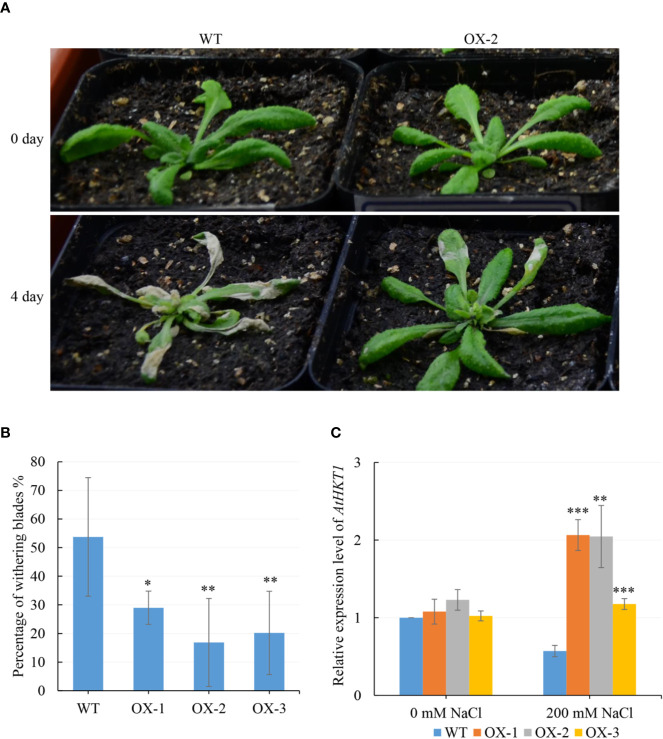
*NtCIPK9* overexpressing homozygous Arabidopsis showed a higher salt tolerance than WT. **(A)** 200 mM NaCl treated T3 transgenic plants and WT for 4 days. **(B)** Percentage of withering blades. **(C)** *AtHKT1* expression of transgenic plants (OX-1, OX-2, OX-3) and WT in pots treated by salt stress. ANOVA test was conducted to determine statistical significance of the results. ***P < 0.001; **P < 0.01; *P < 0.05.

To address whether ectopic expression of *NtCIPK9* could influence salt tolerance of plants, we grew *35S:NtCIPK9* and WT seeds on salt-rich media with 100 and 150 mM NaCl. *35S:NtCIPK9* seedlings showed better growth with more leaves and longer primary root on both media compared to WT plants, 20 days after germination (**Figures 5A–C**). This effect is more clear when plants grew on medium with a higher salt concentration (**Figures 5B, C**). To further assess salt-tolerance of the transgenic plants, 10-day-old seedlings were treated with 150 mM NaCl. 10 days after treatment, plants grown on medium without salt (**Figure 6A**) displayed no different phenotype. However, the number of whitening leaves and the mortality rate in WT were significantly higher than that of three transgenic lines grown on media with 150 mM NaCl (**Figures 6B, C**). In addition, enhanced tolerance to salt was also observed in plants grown in pots. Four weeks-old plants of WT and T2 heterozygous transgenic lines, four in a pot in duplicate, were irrigated with 200 mM NaCl for 4 days. All plants displayed withering blades from first day after salt treatment (**Figures 7A–D**). But the plants overexpressing *NtCIPK9* showed a lower percentage of withering leaves than WT under salt stress (**Figure 7E**). Similarly, four-week-old T3 homozygous transgenic plants in pots also showed a higher salt tolerance than WT under 200 mM NaCl treatment for 4 days (**Figures 8A, B**).

### Ectopic Expression of *NtCIPK9* in Arabidopsis Elevates K^+^ Accumulation

To investigate how ectopic expression of *NtCIPK9* causes increased salt tolerance, we measured the Na^+^ and K^+^ content of *35S:NtCIPK9* transgenic plants under salt stress. Under normal conditions, the ion content of these transgenic lines has no difference with WT. By contrast, although salt stress increased the Na^+^ content of both WT and transgenic plants, transgenic plants do show a slightly lower Na^+^ content than WT (**Figure 5D**). More importantly, salt treatment reduced the K^+^ content of transgenic plants to a lesser extent than that of WT (**Figure 5E**).

**Figure 9:**
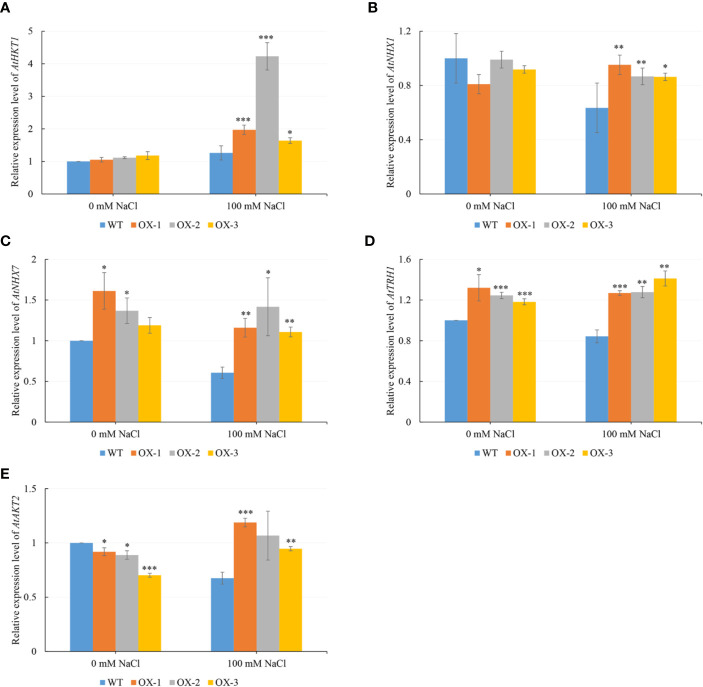
*NtCIPK9* promoted the expression of Na^+^ or/and K^+^ transporter genes. **(A**–**E)** Gene expression level of transgenic plants (OX-1, OX-2, OX-3) and WT treated by 100 mM NaCl on petri dishes for 20 days. Data represent means ± SD from three technical replicates. ANOVA test was used for statistical analysis. ***P < 0.001; **P < 0.01; *P < 0.05.

To figure out what might be causing the difference in Na^+^ and K^+^ content, we analyzed five genes which are known to be involved in Na^+^ or/and K^+^ transportation. We found that *AtHKT1* was around 2-fold upregulated in at least two *NtCIPK9* transgenic plants, compared to wildtype plants treated in pots (**Figure 8C**). Similarly, the *AtHKT1* expression level was also significantly upregulated in the transgenic plants treated on petri dishes (**Figure 9A**). Besides, the expression level of the other four genes (*AtNHX1*, *AtNHX7*, *AtTRH1*, and *AtAKT2*) was higher in the transgenic plants than in WT seedlings under salt stress (**Figures 9B–E**). To further ensure the function of *CIPK*s in salt stress, we checked the transcription of other known *CIPK*s in *Nitraria tangutorum* (*NtCIPK2*) (Zheng L. L. et al., 2014). The results showed that the *CIPK*s were also positively response to salt stress in both *Nitraria tangutorum* and *Arabidopsis thaliana* (**Supplementary Figure 3**).

## Discussion

Salinization of arable land is a serious threat to agricultural and ecological stability. Therefore, halophytes have become promising candidates for further management of salinized areas. In order to adapt to their stressful environment, these plants developed a series of regulatory mechanisms during evolution. At the same time, genetic engineering of glycophytes by transforming genes from halophytes has been widely used to improve their salt resistance (Himabindu et al., 2016). In our study, we found that overexpression of *NtCIPK9* from *Nitraria tangutorum* in Arabidopsis increased seed germination rate under salt stress. There’s one possible reason that could cause the higher seed germination of transgenic plants. *AtCIPK3* from Arabidopsis has been reported to be involved in the phytohormone abscisic acid (ABA) response, which plays a vital role in seed maturation, dormancy, and seed germination (Finkelstein et al., 2002; Pandey et al., 2008b). Therefore, we thought *NtCIPK9* increasing seed germination rate could also be related with plant endogenous ABA. On the media without NaCl, we found the WT has a high germination rate as the *NtCIPK9*-overexpressing seeds. *NtCIPK9* here didn’t show a positive function on seed germination because the germination rate of WT was close to 100% at normal condition. However, *NtCIPK9* effectively enhanced the seed germination under salt treatment, that reflected the function of *NtCIPK9* on coping with salt stress.

*BnCIPK9* from *Brassica napus L*. was reported to regulate seed oil content, a different function than was reported for *Arabidopsis AtCIPK9* (Guo et al., 2018), suggesting that *CIPK* orthologs from different species can also have roles other than only being involved in salt tolerance. However, we found that *NtCIPK9* may share a similar role in regulating ion homeostasis with the ortholog of Arabidopsis (Pandey et al., 2007; Li-Li et al., 2013). *AtCIPK9* has been identified as a critical regulator of potassium transporters in Arabidopsis, that are involved in potassium acquisition, with some of them being critical for potassium nutrition under low potassium conditions (Pandey et al., 2007). In our study, the results also suggested that the overexpression of *NtCIPK9* might regulate the expression of potassium transporter *AtHKT1* to promote the homeostasis of Na^+^ and K^+^ in Arabidopsis resistance for salt stress. This revealed the common identity of orthologs from different species. However, other genes for cation transportation (*AtNHX1*, *AtNHX7*, *AtTRH1* and *AtAKT2*), which have been reported to respond to salinity, were not upregulated even in transgenic plants after salt treatment. But compared with WT, the transgenic plants have a relative higher expression level (**Figures 9B–E**). The possible reason could be that these gene are not the key factors regulated by *NtCIPK9*; or these genes responding to the stress are asynchrony because of the necessity for long-time stress resistance.

## Conclusion

In our research, we identified a novel *CIPK* gene, *NtCIPK9*, which positively responds to salt stress in *Nitraria tangutorum*. Overexpression of *NtCIPK9* in Arabidopsis plants increases seed germination rate, root length, leaf number, and reduces mortality rate under salt stress. Furthermore, *NtCIPK9* may enhance the tolerance of transgenic plants to salinity by increasing the expression level of genes in balancing ion homeostasis after the salt treatment. Altogether, our study revealed that *NtCIPK9* from the halophyte *Nitraria tangutorum* could improve the salt tolerance of Arabidopsis, which would further contribute to the genetic engineering of other glycophytes for stronger salt resistance and sheds light on the molecular mechanism causing the enhanced resistance. However, more practical application of halophytes facing various degree of stresses need to be further investigated.

## Data Availability Statement

The original contributions presented in the study are publicly available. This data can be found here: https://www.ncbi.nlm.nih.gov/nuccore/MN852853.

## Author Contributions

JC and JS contributed conception and design of the study. LZ, ML, JZ, XY, PW, YL, TC, and YY performed the experiments and carried out the statistical analysis. LL and XC wrote sections of the manuscript. All authors contributed to the article and approved the submitted version.

## Funding

This research was supported by Key research and development plan of Jiangsu Province (BE2017376), Foundation of Jiangsu forestry bureau (LYKJ[2017]42), the Nature Science Foundation of China (31770715), the Qinglan project of Jiangsu province, A Project Funded by the Priority Academic Program Development of Jiangsu Higher Education Insitutions (PAPD), Natural Science Foundation of Jiangsu Province (BK20181176) and the Joint Fund of the Natural Science Foundation of China and the Karst Science Research Center of Guizhou Province (Grant No. U1812401).

## Conflict of Interest

The authors declare that the research was conducted in the absence of any commercial or financial relationships that could be construed as a potential conflict of interest.
