# Supplementary figures and images for: NtCIPK9: A Calcineurin B-Like Protein-Interacting Protein Kinase From the Halophyte Nitraria tangutorum, Enhances Arabidopsis Salt Tolerance
Source: Front Plant Sci. 2020 Aug 21;11:1112. doi: 10.3389/fpls.2020.01112 (PMC7472804; doi:10.3389/fpls.2020.01112)

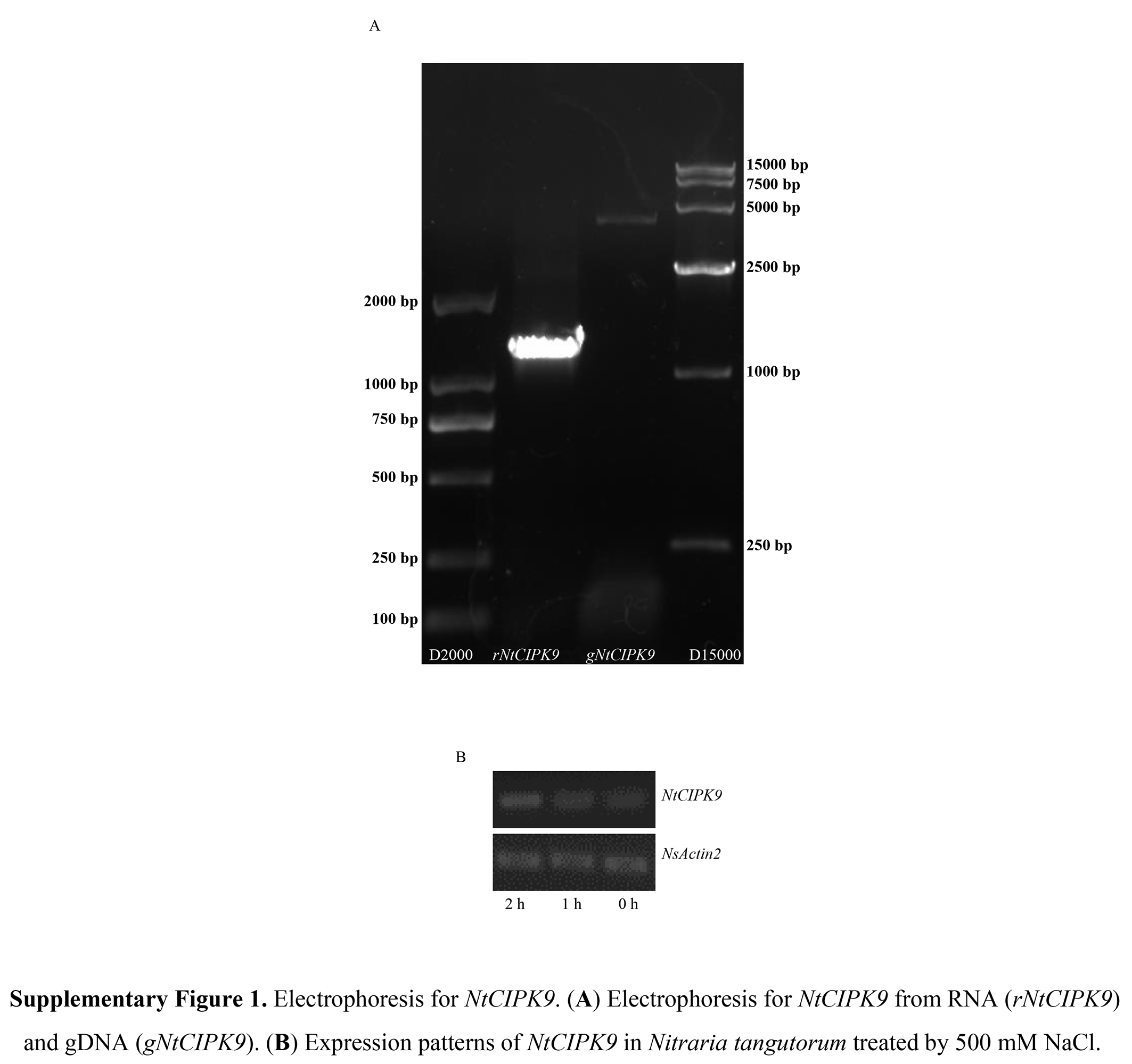

Supplement: Supplementary file 1 [file Image_1.tif]

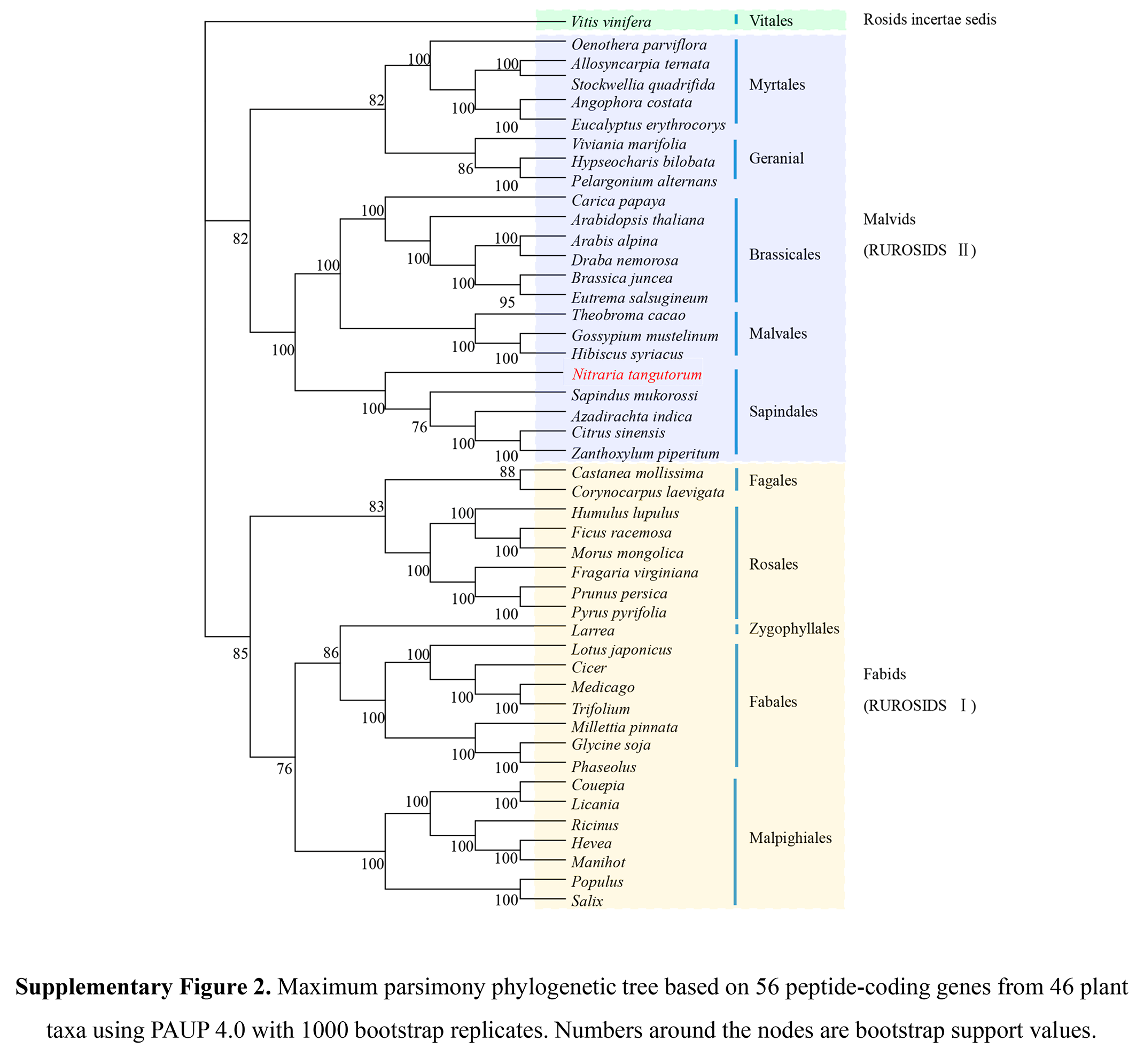

Supplement: Supplementary file 2 [file Image_2.tif]

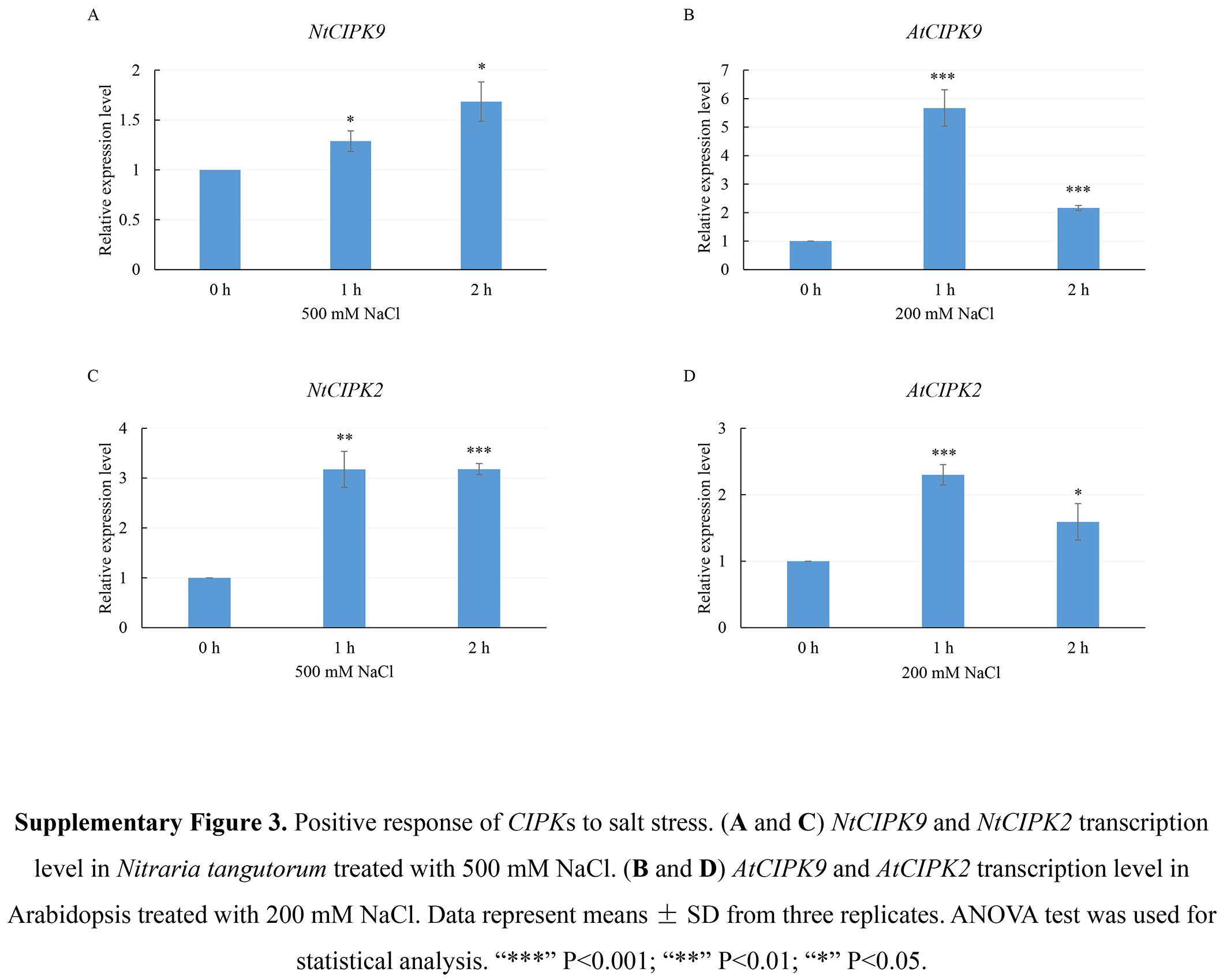

Supplement: Supplementary file 3 [file Image_3.tif]
