# Supplementary material for: NtCIPK9: A Calcineurin B-Like Protein-Interacting Protein Kinase From the Halophyte Nitraria tangutorum, Enhances Arabidopsis Salt Tolerance
Source: Front Plant Sci. 2020 Aug 21;11:1112. doi: 10.3389/fpls.2020.01112 (PMC7472804; doi:10.3389/fpls.2020.01112)
Supplement: Supplementary file 4 [file Table_1.docx]

| Supplementary Table 1. Primers for isolation of *NtCIPK9* fragment | |
| --- | --- |
| Primer name | Sequence (5’-3’) |
| *NtCIPK9* forward primer | GTGATCAAGTCCTGCGTCACAA |
| *NtCIPK9* reverse primer | CTACCTCAAACACCTCAGTGGCTAC |

Supplementary tables

| Supplementary Table 2. Primers for RACE | |
| --- | --- |
| Primer name | Sequence (5’-3’) |
| *CIPK9*:3’race primer A | GTGGATGCCGTTTTCAATGACTCGAAGG |
| *CIPK9*:3’race primer B | CCTCGAGAACTTATTTGAGAAGCAGACGGG |
| *CIPK9*:3’race primer C | GAGAAGCAGACGGGTCTTGTGAAGCGAG |
| *CIPK9*:5’race primer A | CCGCACTTGCTGCGACAGCGCAC |
| *CIPK9*:5’race primer B | TCTGTTCGACCATCTTGTGACGCAGGACTTG |
| *CIPK9*:5’race primer C | CTCCGGTCTCAATCTTGGCGAATTTCACC |

| Supplementary Table 3. Primers for cloning coding region of *NtCIPK9* gene | |
| --- | --- |
| Primer name | Sequence (5’-3’) |
| *CIPK9*:wl forward primer | GGATCCATGAATAAGGTACCGGGGAC |
| *CIPK9*:wl reverse primer | CCCGGGCGTGATTTCTTTACAGC |

Supplementary Table 4. *CIPK* family genes in Arabidopsis for phylogenic analysis

| Gene name | Accession number |
| --- | --- |
| *AtCIPK1* | AAG28776.1 |
| *AtCIPK2* | AAF86506.1 |
| *AtCIPK3* | AEC07917.1 |
| *AtCIPK4* | AAG01367.1 |
| *AtCIPK5* | AAF86504.2 |
| *AtCIPK6* | AEE85835.1 |
| *AtCIPK7* | AEE76704.1 |
| *AtCIPK8* | AEE84900.1 |
| *AtCIPK9* | AEE27245.1 |
| *AtCIPK10* | AED97046.1 |
| *AtCIPK11* | AAK16686.1 |
| *AtCIPK12* | AEE84078.1 |
| *AtCIPK13* | AEC08929.1 |
| *AtCIPK14* | AED90397.1 |
| *AtCIPK15* | AED90395.1 |
| *AtCIPK16* | AAK50348.1 |
| *AtCIPK17* | AAK64513.1 |
| *AtCIPK18* | AAK59695.1 |
| *AtCIPK19* | AAK50347.1 |
| *AtCIPK20* | AED95303.1 |
| *AtCIPK21* | AAK59696.1 |
| *AtCIPK22* | AEC09543.1 |
| *AtCIPK23* | AAK61494.1 |
| *AtCIPK24* | AED93966.1 |
| *AtCIPK25* | AED93402.1 |
| *AtCIPK26* | AED92948.1 |

Supplementary Table 5. List of chloroplast genome sequence accession numbers used in the phylogenetic study.

| No. | Taxon | Order | GenBank accession number |
| --- | --- | --- | --- |
| 1 | *Pyrus pyrifolia* | Rosales | NC_015996.1 |
| 2 | *Prunus persica* | Rosales | NC_014697.1 |
| 3 | *Fragaria virginiana* | Rosales | NC_019602.1 |
| 4 | *Humulus lupulus* | Rosales | NC_028032.1 |
| 5 | *Ficus racemosa* | Rosales | NC_028185.1 |
| 6 | *Morus mongolica* | Rosales | NC_025772.2 |
| 7 | *Castanea mollissima* | Fagales | NC_014674.1 |
| 8 | *Corynocarpus laevigata* | Fagales | NC_014807.1 |
| 9 | *Glycine soja* | Fabales | NC_022868.1 |
| 10 | *Phaseolus vulgaris* | Fabales | NC_009259.1 |
| 11 | *Millettia pinnata* | Fabales | NC_016708.2 |
| 12 | *Lotus japonicus* | Fabales | NC_002694.1 |
| 13 | *Cicer arietinum* | Fabales | NC_011163.1 |
| 14 | *Trifolium subterraneum* | Fabales | NC_011828.1 |
| 15 | *Medicago papillosa* | Fabales | NC_027154.1 |
| 16 | *Larrea tridentata* | Zygophyllales | NC_028023.1 |
| 17 | *Manihot esculenta* | Malpighiales | NC_010433.1 |
| 18 | *Hevea brasiliensis* | Malpighiales | NC_015308.1 |
| 19 | *Ricinus communis* | Malpighiales | NC_016736.1 |
| 20 | *Couepia guianensis* | Malpighiales | NC_024063.1 |
| 21 | *Licania sprucei* | Malpighiales | NC_024065.1 |
| 22 | *Salix purpurea* | Malpighiales | NC_026722.1 |
| 23 | *Populus trichocarpa* | Malpighiales | NC_009143.1 |
| 24 | *Eucalyptus erythrocorys* | Myrtales | NC_022406.1 |
| 25 | *Angophora costata* | Myrtales | NC_022412.1 |
| 26 | *Stockwellia quadrifida* | Myrtales | NC_022414.1 |
| 27 | *Allosyncarpia ternata* | Myrtales | NC_022413.1 |
| 28 | *Oenothera parviflora* | Myrtales | NC_010362.1 |
| 29 | *Viviania marifolia* | Geraniales | NC_023259.1 |
| 30 | *Hypseocharis bilobata* | Geraniales | NC_023260.1 |
| 31 | *Pelargonium alternans* | Geraniales | NC_023261.1 |
| 32 | *Zanthoxylum piperitum* | Sapindales | NC_027939.1 |
| 33 | *Citrus sinensis* | Sapindales | NC_008334.1 |
| 34 | *Azadirachta indica* | Sapindales | NC_023792.1 |
| 35 | *Sapindus mukorossi* | Sapindales | NC_025554.1 |
| 36 | *Nitraria tangutorum* | Sapindales | MK347423.1 |
| 37 | *Gossypium mustelinum* | Malvales | NC_016711.1 |
| 38 | *Hibiscus syriacus* | Malvales | NC_026909.1 |
| 39 | *Theobroma cacao* | Malvales | NC_014676.2 |
| 40 | *Carica papaya* | Brassicales | NC_010323.1 |
| 41 | *Arabidopsis thaliana* | Brassicales | NC_000932.1 |
| 42 | *Eutrema salsugineum* | Brassicales | NC_028170.1 |
| 43 | *Brassica juncea* | Brassicales | NC_028272.1 |
| 44 | *Arabis alpina* | Brassicales | NC_023367.1 |
| 45 | *Draba nemorosa* | Brassicales | NC_009272.1 |
| 46 | *Vitis vinifera* | Vitales | NC_007957.1 |

| Supplementary Table 6. Primers for subcellular analysis | |
| --- | --- |
| Primer name | Sequence (5’-3’) |
| *CIPK9*:GFP:F | CCCAAGCTTATGAATAAGGTACCGGGGAC |
| *CIPK9*:GFP:R | CGCGGATCCACGTGATTTCTTTACAGCTTTTTC |

| Supplementary Table 7. Primers used for genes related to ion homeostasis in Arabidopsis for real time-PCR analysis. | | | |
| --- | --- | --- | --- |
| Gene name | Accession number | Primer name | Sequence (5’-3’) |
| *NtCIPK9* | MN852853 | *NtCIPK*9F | TGGATGCCGTTTTCAATGACTCG |
|  |  | *NtCIPK9*R | GCTTCACAAGACCCGTTTGCTTCT |
| *NsActin2* | AB617805 | *NsActin*F | CATCCCTCATCGGAATGGAAGC |
|  |  | *NsActin*R | GGTAGACCCACCACTAAGCACAATG |
| *AtAKT2* | AT4G22200.1 | *AtAKT2* F | TGAATCTCCTGGCGACGAAC |
|  |  | *AtAKT2* R | CTGTAAATACTCACTCTTCCTCGTT |
| *AtHKT1* | AT4G10310.1 | *AtHKT1* F | CTCATTTCAATCACCGAAAGGC |
|  |  | *AtHKT1* R | TCCATATGCACTGATAACTTCGAGA |
| *AtNHX1* | AT5G27150.1 | *AtNHX1* F | TACACATGGCACAATGTAACGG |
|  |  | *AtNHX1* R | CTCACGGATCTCCACTTGTCA |
| *AtNHX7* | AT2G01980.1 | *AtNHX7* F | TAAAGAAGGTTCAAAGCCAACTGGA |
|  |  | *AtNHX7* R | TAGAGTCCCAATGTACTACCGTGA |
| *AtTRH1* | AT4G23640.1 | *AtTRH1* F | ATCATCAACTTTGTCTGGAACCGTA |
|  |  | *AtTRH1* R | TTTGACTAAGGCTGATGCAACGAA |
| *AtUBQ10* | At4g05320.2 | *AtUBQ10* F | CCGGAAAGACCATCACCCTTG |
|  |  | *AtUBQ10* R | TGTAGTCGGCCAAAGTACGTC |
| *NtCIPK2* | KC823044.1 | *NtCIPK2 F* | ACGACACTTCCTGCCTCAAATA |
|  |  | *NtCIPK2 R* | CCCAAACAATGTCCTCCAGAGC |
| *AtCIPK9* | AT1G01140.3 | *AtCIPK9* F | CTTAAGGAGGATGAAGCTCGGAG |
|  |  | *AtCIPK9* R | GAACTTGTCGTGAGAAGGCGCTT |
| *AtCIPK2* | AT5G07070.1 | *AtCIPK2* F | CTGCCAGACCAAACCCCGTA |
|  |  | *AtCIPK2* R | TTCAGGCCAAAATGAAAACGGAGA |
